# Non-linear associations of total and leisure-time physical activity with chronic kidney disease: Findings from NHANES

**DOI:** 10.1371/journal.pone.0334224

**Published:** 2025-10-08

**Authors:** Chuixuan Zeng, Qin Lang, Keqin Zhang, Xiuzhen Qian

**Affiliations:** 1 Department of Urinary Nephropathy Center, The Second Affiliated Hospital of Chongqing Medical University, Chongqing, China; 2 Department of Clinical Nutrition, Chongqing Red Cross Hospital (People’s Hospital of Jiangbei District), Chongqing, China; 3 Department of General Surgery, The Fifth People’s Hospital of Chongqing, Chongqing, China; Florida State University, UNITED STATES OF AMERICA

## Abstract

Physical activity (PA) is a protective factor for many chronic diseases, but its association with chronic kidney disease (CKD) remains unclear. Using National Health and Nutrition Examination Survey (NHANES) data (2007–2016), this study examined the relationship between PA and CKD among participants aged 20 years or older who had valid data on both kidney function and PA. PA was assessed via the Global Physical Activity Questionnaire, which captured the frequency, duration, and intensity of occupation-activities, transportation-activities, and leisure-time-related activities. CKD was defined using estimated glomerular filtration rate (eGFR) calculated from serum creatinine and urinary albumin-to-creatinine ratio (UACR), based on KDIGO guidelines. Multivariable logistic regression revealed a significant inverse association between total PA and CKD risk (OR = 0.90; 95% CI, 0.84–0.96; P = 0.003), and leisure-time PA (LTPA) was also inversely associated with CKD (OR = 0.89; 95% CI, 0.82–0.96; P = 0.002), while occupation-related PA (OPA) and transportation-related PA (TPA) showed no significant associations. Subgroup and interaction analyses of total PA and LTPA across survey cycles, sex, age, race, educational level, FPIR, marital status, smoking status, drinking status, dietary supplements and protein, total fat, carbohydrate and energy from diet identified no significant effect modifiers. We further performed restricted cubic spline (RCS) analyses for both total PA and LTPA, which revealed significant non-linear inverse relationships with CKD risk (all P < 0.001 for non-linearity). In addition, mediation analyses suggested that the inverse association between total PA and CKD was partially mediated through BMI, diabetes, hypertension, and systemic inflammation.

## 1. Introduction

Over the period from 1990 to 2017, the global prevalence of chronic kidney disease (CKD) has increased 29.3%, driven by rising rates of major risk factors like an aging population, diabetes, obesity, infection, and hypertension [1]. With its hallmarks of irreversibly progressive kidney damage and impaired function, CKD has become a global public health issue [2,3]. A major societal effect of CKD is the immense healthcare costs associated with its potential outcome, end-stage renal disease, and patients can die or receive costly renal replacement therapy, posing a massive health and economic burden [4,5]. Hence, ascertaining modifiable risk factors is of critical importance for the prevention and mitigation of CKD risk.

Regular physical activity (PA), comprising three domain-specific PAs, occupation-related PA (OPA), transportation-related PA (TPA), and leisure-time PA (LTPA), is widely recognized as mandatory for the prevention and treatment of obesity, diabetes, hypertension, and insulin resistance, which have shared risk factor profiles with CKD [6,7]. In addition to these effects, PA plays a crucial role in glycemic control and weight regulation. It enhances insulin sensitivity [8–10], lowers fasting glucose levels [11], and reduces visceral fat accumulation [12]—all of which contribute to mitigating the harmful impact of diabetes and obesity on kidney function. Moreover, accumulating evidence indicates that regular PA effectively suppresses systemic inflammation [13–15], a key pathophysiological mechanism implicated in the development and progression of CKD [16–18]. Through these interrelated mechanisms—modulating traditional metabolic risk factors and alleviating chronic inflammation—PA is hypothesized to lower the risk of CKD onset.

Several studies have provided supporting evidence for this hypothesis. For example, a large Taiwanese cohort study using generalized linear mixed models (GLMM) and Cox proportional hazards regression—adjusting for age, sex, education, smoking and alcohol use, occupational PA, diet, BMI, and comorbid hypertension, diabetes, cardiovascular disease, and cancer—found that higher self-reported weekly habitual physical activity was associated with slower eGFR decline and lower CKD risk [19]. Similarly, in the Atherosclerosis Risk in Communities (ARIC) study (n = 14,537; median follow-up 24 years), Parvathaneni et al. similarly observed an inverse relationship between both baseline and time-updated PA and incident CKD: after full adjustment for covariates, the highly active group had an HR of 0.89 (95% CI 0.81–0.97; P-trend = 0.007) compared with the inactive group. When PA was modeled as a time-varying covariate, the HR for the highly active group further decreased to 0.83 (95% CI 0.74–0.92; P-trend < 0.001) [20]. However, not all studies have reached consistent conclusions. For instance, Hawkins et al. conducted a prospective cohort study and found no significant association between PA and either incident CKD or rapid kidney function decline [21]. Likewise, Zhu and colleagues, in a recent meta-analysis of eight cross-sectional studies, reported little evidence of an association between the highest versus lowest PA levels and CKD risk [22]. These findings suggest that the relationship between PA and CKD risk may vary depending on population characteristics, study design, and the methods used to assess PA. Further research, particularly prospective and interventional studies, is warranted to clarify PA’s mechanisms in CKD prevention and to define optimal activity prescriptions.

Despite existing studies exploring the relationship between PA and CKD risk, the results may not be universally applicable to the entire U.S. population, as they often fail to account for the broad diversity. Therefore, this study aims to utilize data from the National Health and Nutrition Examination Survey (NHANES) to investigate the detailed relationship between PA and CKD risk. NHANES employs stratified multistage sampling techniques to obtain nationally representative data, making the research findings more generalizable and reliable for the overall U.S. population [23]. By adopting this approach, we hope to address gaps in the current literature and provide new insights for public health policies and preventive strategies.

## 2. Methods

### 2.1. Study population

NHANES is an ongoing cross-sectional survey conducted by the National Center for Health Statistics of the Centers for Disease Control and Prevention [23]. Beginning in 1999, NHANES transitioned to a continuous annual data collection process with findings released in 2-year cycles. The survey combines in-home interviews and standardized physical examinations performed by trained staff in mobile examination centers. Additional details about methodology and data releases are available on the NHANES website (https://wwwn.cdc.gov/nchs/nhanes/). As this study conducted a secondary analysis of publicly available NHANES data, institutional review board approval was not required.

**Fig 1:**
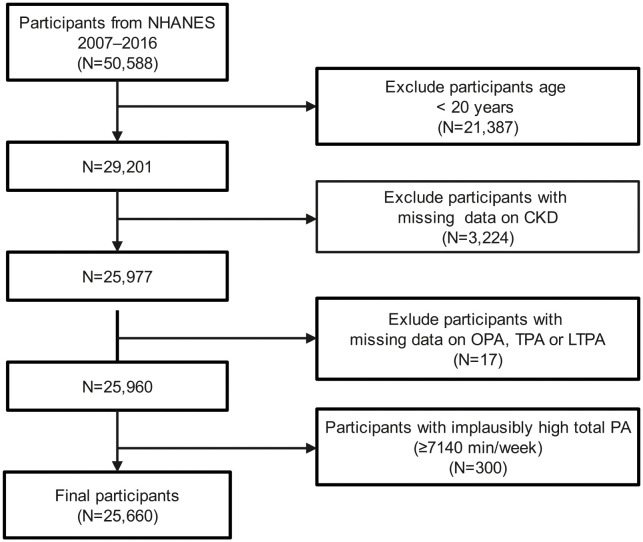
Flowchart of participant selection from NHANES 2007–2016. Participants aged 20 years and older from NHANES 2007–2016 were initially considered (n = 29,201). Individuals with missing data on kidney function or physical activity (n = 3,241) and those with implausible physical activity values (n = 300) were excluded. Multiple imputation was performed for participants with missing demographic, clinical, or dietary variables. A total of 25,660 participants were included in the final analysis.

Data used in this study were obtained from five NHANES cycles (2007–2008, 2009–2010, 2011–2012, 2013–2014, and 2015–2016), following the analytical guidelines. Participants aged 20 years and older were enrolled (n = 29,201), similar to other studies [24,25]. As shown in Fig 1, we first excluded participants with missing key data on kidney function or physical activity (n = 3,241). In addition, participants with implausible physical activity data were excluded (n = 300), based on the assumption that healthy adults should sleep at least 7 hours per day, as recommended by U.S. guidelines [26]. For participants with missing demographic, medical, or dietary variables—including sex, age, race, educational level, family poverty income ratio (FPIR), marital status, smoking status, drinking status, body mass index (BMI), hypertension, diabetes, dietary supplements, and protein, total fat, carbohydrate and energy from diet—we performed multiple imputation to address missingness. After exclusions, a total of 25,660 participants were included in the final analysis.

### 2.2. Physical activity

Physical activity was self-reported by participants using the Global Physical Activity Questionnaire [27], which collected the frequency (time per week), duration (minutes per time), and intensity (vigorous or moderate) of three domain-specific PAs (OPA, TPA, and LTPA) in one typical week. As previous research indicates the effect of vigorous PA is twice that of moderate PA [28], the minutes of vigorous PA performed by each participant were multiplied by 2 and then combined with the minutes of moderate PA for that individual. In the current study, total PA was categorized into two groups based on whether participants met the 2018 PA guidelines [29], which recommend that adults should engage in at least 150–300 min per week of moderate-intensity PA, 75–150 min per week of vigorous-intensity PA, or an equivalent combination. Furthermore, to assess the dose-response relationship between total PA and LTPA with CKD risk, we categorized total PA and LTPA based on the 25th, 50th, and 75th percentiles of their distributions. Specifically, total PA was divided into four groups: (1) 0 min/week, (2) 1–240 min/week, (3) 240–899 min/week, (4) ≥900 min/week. Given the considerable bias in LTPA data, the LTPA minutes were categorized into three groups: (1) 0 min/week, (2) 1–239 min/week, (3) ≥240 min/week.

### 2.3. Confirmation of CKD

Serum creatinine, urinary creatinine, and urinary albumin values were obtained from the laboratory data available in NHANES. Serum creatinine was measured using the Jaffé rate reaction and were converted to estimated glomerular filtration rate (eGFR) using the Chronic Kidney Disease Epidemiology Collaboration [30]. The measurement of urinary albumin and creatinine used a solid-phase fluorescent immunoassay and a Jaffé rate reaction, respectively. Participants diagnosed with CKD were those with an eGFR less than 60 mL/min/1.73m^2^ or urinary albumin-to-creatinine ratio (UACR) greater than or equal to 30 mg/g according to the Kidney Disease Improving Global Outcomes guidelines [31].

### 2.4. Control of confounders

Sociodemographic information, including age, sex, race, educational level, marital status, and FPIR, was collected during in-home interviews conducted by trained interviewers. Survey cycles were included as part of the sociodemographic dataset. The FPIR, calculated by dividing family income by poverty guidelines specific to family size, year, and state, was based on the Department of Health and Human Services’ poverty measure. FPIR was then categorized into three groups: low (≤1), middle (1 < FPIR ≤ 4), and high (FPIR > 4) [32,33]. Data on lifestyle habits (smoking and drinking status) and prevalence of diseases (hypertension and diabetes) were obtained from Household Interview Component Questionnaires. Body mass index (BMI, kg/m^2^) was calculated as weight divided by height squared, with both weight and height measured while participants were wearing light clothing without shoes. Additionally, dietary information on protein, total fat, carbohydrate and energy intake from foods and use of dietary supplements was collected based on two 24-hour dietary recall periods. The first dietary recall was collected in-person during the NHANES visit, while the second was collected by telephone 3 to 10 days later. For subsequent analyses, the protein, total fat, carbohydrate, and energy intake values were averaged over the two 24-hour recall periods. If only the first day of recall was available, the solitary value was used instead of an average.

To guide variable selection and enhance the rigor of causal inference, we constructed a directed acyclic graph (DAG) (S1 Fig) to represent the hypothesized relationship between PA and CKD, informed by previous literature [13,18,34–46]. Based on the DAG structure, we identified the minimal sufficient adjustment set to control for confounding, including survey cycles, sex, age, race, educational level, FPIR, marital status, smoking status, drinking status, dietary supplements, and dietary intake of protein, total fat, carbohydrate, and energy. These variables were adjusted for in all multivariable models.

Considering that body mass index (BMI), diabetes, hypertension, and inflammation may lie on the causal pathway between PA and CKD, they were treated as potential mediators and assessed separately through mediation analysis. Based on prior studies, white blood cell (WBC) count, lymphocyte count, absolute neutrophil count, and alkaline phosphatase (ALP) were selected as inflammatory markers. These biomarkers have been widely used in NHANES-based research to assess systemic inflammation [47,48].

### 2.5. Statistical analysis

Baseline characteristic data were grouped by CKD diagnosis and presented as weighted mean and weighted standard deviation (SD) for continuous variables, and unweighted counts and weighted percentages for categorical variables. Group differences were obtained using t-tests for continuous variables and chi-squared tests for categorical variables. To evaluate the association of PA, OPA, TPA, and LTPA with CKD, logistic regression analyses were conducted using two multivariate models. Model 1 was adjusted by survey cycles, sex, age, race, educational level, FPIR and marital status. Model 2 was further adjusted by smoking status, drinking status, dietary supplements, and protein, total fat, carbohydrate, and energy from diet. Additionally, we employed restricted cubic spline (RCS) models to describe the relationship between CKD risk and both total PA and LTPA across their full range [49].

To assess the consistency of associations between total PA and LTPA with CKD risk across various subgroups, we conducted both stratified analyses and interaction analyses. Specifically, subgroup analyses were performed according to survey cycles (2007–2010 vs. 2011–2016), age (>65 vs. ≤ 65 years), sex (male vs. female), race (Hispanic vs. Non-Hispanic), educational level (less than college vs. some college or above), family poverty income ratio (high vs. middle vs. low), marital status (living alone vs. living with others), smoking status (never vs. current vs. former), drinking status (never vs. current vs. former), and energy intake (≤median vs. > median). Additionally, to evaluate whether these covariates modified the association between PA and CKD risk, interaction terms (e.g., PA × age, LTPA × age) were included in multivariable logistic regression models, and corresponding P values for interaction were reported to assess potential effect modification.

Additionally, mediation analyses were performed in the fully adjusted model (Model 2) using 1,000 bootstrap resamples to assess whether BMI, diabetes, hypertension, and inflammation mediated the relationship between total PA and CKD risk [50–53].

The overall data analysis process is summarized in S2 Fig. All statistical analyses were adjusted for the complex survey designs of NHANES. The statistical significance level was set at a P value of < 0.05. All analyses were performed using R 4.3.1 software.

## 3. Results

### 3.1. Baseline table

After excluding ineligible participants, 25,660 individuals remained for further analysis across five cycles. Of the 25,660 participants in the final analytic cohort, 14% (n = 4,570) were diagnosed with CKD, and their characteristics, stratified by CKD diagnosis, are presented in Table 1. Substantial differences in participant characteristics were observed between the two groups. Compared to participants without a CKD diagnosis, CKD patients exhibited increased proportions of females, lower physical activity, older age, Non-Hispanic Black and Non-Hispanic White ethnicities, lower educational levels, middle FPIR, living together, higher BMI, being never smokers and current drinkers, and having hypertension and not having diabetes. Furthermore, in terms of dietary habits, CKD patients tended to have a diet that was lower in energy, protein, fat, and carbohydrates, while showing a preference for dietary supplements.

**Table 1:** Baseline characteristics in all qualified NHANES 2007-2016 participants.

		Diagnosed as CKD		
Characteristic	Overall, N = 25660 (100%)	No, N = 21090 (86%)	Yes, N = 4570 (14%)	P Value
Physical activity	818.52(1,220.00)	865.79(1,247.04)	533.26(995.62)	<0.001
Sex				<0.001
Male	12,393.00(48.00%)	10,245.00(48.94%)	2,148.00(42.31%)	
Female	13,267.00(52.00%)	10,845.00(51.06%)	2,422.00(57.69%)	
Age (years)	47.38(16.79)	45.18(15.66)	60.66(17.22)	<0.001
Race				<0.001
Mexican American	3,997.00(8.56%)	3,373.00(8.75%)	624.00(7.39%)	
Non-Hispanic Black	5,175.00(10.67%)	4,168.00(10.44%)	1,007.00(12.08%)	
Non-Hispanic White	10,856.00(67.41%)	8,711.00(67.08%)	2,145.00(69.40%)	
Other Hispanic	2,770.00(5.68%)	2,355.00(5.85%)	415.00(4.69%)	
Other Race	2,862.00(7.68%)	2,483.00(7.88%)	379.00(6.44%)	
Educational level				<0.001
Less than college	12,236.00(38.42%)	9,651.00(36.83%)	2,585.00(47.96%)	
Some college or above	13,424.00(61.58%)	11,439.00(63.17%)	1,985.00(52.04%)	
FPIR				<0.001
High	6,370.00(35.69%)	5,514.00(37.06%)	856.00(27.45%)	
Low	5,833.00(15.55%)	4,707.00(15.09%)	1,126.00(18.34%)	
Middle	13,457.00(48.75%)	10,869.00(47.85%)	2,588.00(54.21%)	
Marital status				<0.001
Live alone	10,306.00(36.18%)	8,203.00(35.22%)	2,103.00(41.96%)	
Live together	15,354.00(63.82%)	12,887.00(64.78%)	2,467.00(58.04%)	
BMI	28.92(6.74)	28.75(6.61)	29.95(7.42)	<0.001
Smoking status				<0.001
Current	5,251.00(19.89%)	4,452.00(20.36%)	799.00(17.04%)	
Former	6,118.00(24.52%)	4,632.00(23.16%)	1,486.00(32.73%)	
Never	14,291.00(55.59%)	12,006.00(56.48%)	2,285.00(50.23%)	
Drinking status				<0.001
Current	18,250.00(76.71%)	15,310.00(78.13%)	2,940.00(68.15%)	
Former	3,495.00(11.49%)	2,724.00(10.79%)	771.00(15.74%)	
Never	3,915.00(11.80%)	3,056.00(11.08%)	859.00(16.11%)	
Hypertension				<0.001
No	16,485.00(68.24%)	14,811.00(72.72%)	1,674.00(41.16%)	
Yes	9,175.00 (31.76%)	6,279.00 (27.28%)	2,896.00 (58.84%)	
Diabetes				<0.001
No	22,417.00 (90.67%)	19,238.00 (93.35%)	3,179.00 (74.46%)	
Yes	3,243.00 (9.33%)	1,852.00 (6.65%)	1,391.00 (25.54%)	
Energy from Diet (kcal/day)	2,109.37 (846.68)	2,146.57 (854.87)	1,884.81 (757.68)	<0.001
Protein from Diet (g/day)	82.69 (36.27)	84.14 (36.57)	73.98 (33.10)	<0.001
Total fat from Diet (g/day)	80.72 (39.40)	82.12 (39.88)	72.25 (35.17)	<0.001
Carbohydrate from Diet (g/day)	252.82 (109.03)	256.98 (110.29)	227.69 (97.39)	<0.001
Dietary supplements				<0.001
No	15,494.00 (57.28%)	13,169.00 (59.04%)	2,325.00 (46.67%)	
Yes	10,166.00 (42.72%)	7,921.00 (40.96%)	2,245.00 (53.33%)	
UACR (mg/g)	33.38 (283.85)	7.80 (5.38)	187.75 (734.14)	<0.001
CKD stage				<0.001
1	15,478.00 (60.80%)	14,145.00 (65.76%)	1,333.00 (30.85%)	
2	7,988.00 (32.49%)	6,945.00 (34.24%)	1,043.00 (21.93%)	
3	1,959.00 (6.14%)	0.00 (0.00%)	1,959.00 (43.17%)	
4	172.00 (0.44%)	0.00 (0.00%)	172.00 (3.08%)	
5	63.00 (0.14%)	0.00 (0.00%)	63.00 (0.97%)	
Survey cycle (year)				0.2
2007–2008	5,119.00 (18.87%)	4,131.00 (18.88%)	988.00 (18.82%)	
2009–2010	5,568.00 (19.59%)	4,634.00 (19.92%)	934.00 (17.62%)	
2011–2012	4,788.00 (19.94%)	3,932.00 (19.96%)	856.00 (19.83%)	
2013–2014	5,191.00 (20.71%)	4,265.00 (20.38%)	926.00 (22.67%)	
2015–2016	4,994.00 (20.89%)	4,128.00 (20.87%)	866.00 (21.06%)	

Categorical variables are presented as unweighted counts and weighted percentages, and continuous variables as weighted means and weighted standard deviations (SD).

All estimates were weighted to account for the complex, multistage sampling design of NHANES.

Abbreviations: FPIR, family poverty income ratio; BMI, body mass index.

### 2.2. Relationship between PA and CKD

As shown in S1 Table, both total physical activity (PA) and leisure-time physical activity (LTPA) were significantly associated with a reduced risk of chronic kidney disease (CKD), whereas no significant associations were observed for occupation-related PA (OPA) or transportation-related PA (TPA), even after adjusting for a comprehensive set of confounders including survey cycles, sex, age, race, educational level, family poverty income ratio (FPIR), marital status, smoking status, drinking status, dietary supplement use, and dietary intake of protein, total fat, carbohydrate, and total energy. Based on these findings, the present study focused primarily on the associations of total PA and LTPA with CKD risk.

Multivariable weighted logistic regression analyses consistently demonstrated inverse associations between both total PA and LTPA with CKD risk after partial and full adjustment for covariates (Table 2 and 3). Specifically, in fully adjusted models (Model 2), each standard deviation (SD) increase in total PA and LTPA was associated with 10% (OR = 0.90; 95% CI: 0.84–0.96; P = 0.003) and 11% (OR = 0.89; 95% CI: 0.82–0.96; P = 0.002) lower odds of CKD, respectively.

**Table 2:** Association between total PA and odds of CKD in all qualified NHANES 2007-2016 participants.

		Unadjusted		Model 1^a^		Model 2^b^	
Total PA (min/week)	Event/n (%)	OR (95% CI)	p-value	OR (95% CI)	p-value	OR (95% CI)	p-value
Per SD increment	4570/25660 (14%)	0.69(0.64, 0.74)	<0.001	0.89(0.83, 0.96)	0.001	0.90(0.84, 0.96)	0.003
Total PA met guidelines							
No	13,143/21,090(89.41%)	1 (Ref)	1 (Ref)	1 (Ref)	1 (Ref)	1 (Ref)	1 (Ref)
Yes	1,972/4,570(10.59%)	0.46(0.42, 0.50)	<0.001	0.71(0.64, 0.79)	<0.001	0.72(0.65, 0.80)	<0.001
Four groups of total PA							
Group 1	1,912/6,960 (23.12%)	1 (Ref)	1 (Ref)	1 (Ref)	1 (Ref)	1 (Ref)	1 (Ref)
Group 2	1,000/5,400 (15.50%)	0.61(0.54, 0.69)	<0.001	0.79(0.69, 0.91)	0.001	0.80(0.70, 0.92)	0.001
Group 3	937/6,689 (11.27%)	0.42(0.37, 0.48)	<0.001	0.67(0.59, 0.77)	<0.001	0.68(0.59, 0.77)	<0.001
Group 4	721/6,611 (9.01%)	0.33(0.29, 0.37)	<0.001	0.65(0.56, 0.75)	<0.001	0.66(0.57, 0.76)	<0.001
P _trend_		<0.001		<0.001		<0.001	

^a^Adjusted for survey cycles, sex, age, race, educational level, FPIR and marital status.

^b^Adjusted for model 1 plus smoking status, drinking status, dietary supplements, and protein, total fat, carbohydrate and energy from diet.

All data was weighted analysis based on the complex survey design of NHANES.

**Table 3:** Association between LTPA and odds of CKD in all qualified NHANES 2007-2016 participants.

		Unadjusted		Model 1^a^		Model 2^b^	
LTPA (min/week)	Event/n(%)	OR (95% CI)	p-value	OR (95% CI)	p-value	OR (95% CI)	p-value
Per SD increment	4570/25660 (14%)	0.67(0.62, 0.73)	<0.001	0.87 (0.81, 0.94)	0.001	0.89(0.82, 0.96)	0.002
Four groups of LTPA							
Group 1	3,011/13,504(18.30%)	1 (Ref)	1 (Ref)	1 (Ref)	1 (Ref)	1 (Ref)	1 (Ref)
Group 2	883/5,625 (16.47%)	0.69(0.61, 0.78)	<0.001	0.89(0.78, 1.02)	0.091	0.89(0.78, 1.02)	0.104
Group 3	676/6,531 (8.49%)	0.41(0.37, 0.46)	<0.001	0.72(0.63, 0.82)	<0.001	0.72(0.63, 0.83)	<0.001
P _trend_		<0.001		<0.001		<0.001	

^a^Adjusted for survey cycles, sex, age, race, educational level, FPIR and marital status.

^b^Adjusted for model 1 plus smoking status, drinking status, dietary supplements, and protein, total fat, carbohydrate and energy from diet.

All data was weighted analysis based on the complex survey design of NHANES.

Moreover, participants who met the recommended total PA guidelines had significantly lower odds of CKD compared with those who did not (OR = 0.72; 95% CI: 0.65–0.80; P < 0.001). When total PA was categorized into quartiles, participants in the highest quartile (Q4) had a substantially lower risk of CKD compared to those in the lowest quartile (Q1) after full covariate adjustment (OR_Q4:Q1_ = 0.66; 95% CI: 0.57–0.76; P < 0.001), with a statistically significant linear trend (P for trend < 0.001). Similarly, when stratified by LTPA quartiles, those in the highest group (Q3) showed a significantly lower risk of CKD compared to the lowest group (Q1) (OR_Q3:Q1_ = 0.72; 95% CI: 0.63–0.83; P < 0.001), also with a significant trend (P for trend < 0.001).

### 3.3. Subgroup and interaction analyses

The results of the subgroup and interaction analyses for total PA and LTPA with CKD risk are presented in Tables 4 and 5, respectively. For both total PA and LTPA, no significant interactions were found across various subgroups, including survey cycles, sex, age, race, educational level, FPIR, marital status, smoking status, drinking status, dietary supplement use, and intake of protein, total fat, carbohydrate, and energy (all interaction P-values > 0.05).

**Table 4:** Subgroup and interaction analyses of the association between total PA and CKD risk in all qualified NHANES 2007-2016 participants.

		Four groups of total PA						
Subgroup variable	Event/n (%)	Group 1	Group 2	Group 3	Group 4	P _trend_	Raw P _interaction_	FDR-adjusted P _interaction_
survey cycles							0.346	0.432
2007–2010	1922/10687 (13.5%)	1 (Ref)	0.76 (0.59, 0.99)	0.76 (0.59, 0.99)	0.59 (0.46, 0.75)	<0.001		
2011-2016	2648/14973 (14.7%)	1 (Ref)	0.82 (0.69, 0.98)	0.68 (0.57, 0.82)	0.70 (0.57, 0.85)	<0.001		
Sex:							0.137	0.432
Male	2148/12393 (12.5%)	1 (Ref)	0.69 (0.56, 0.84)	0.65 (0.54, 0.79)	0.62 (0.51, 0.75)	<0.001		
Female	2422/13267 (15.8%)	1 (Ref)	0.87 (0.74, 1.01)	0.68 (0.58, 0.81)	0.71 (0.59, 0.86)	<0.001		
Age:							0.658	0.658
≤65	2166/20085 (8.9%)	1 (Ref)	0.81 (0.67, 0.99)	0.62 (0.52, 0.74)	0.58 (0.48, 0.70)	<0.001		
>65	2404/5575 (43.1%)	1 (Ref)	0.71 (0.58, 0.86)	0.56 (0.46, 0.67)	0.50 (0.40, 0.64)	<0.001		
Race:							0.335	0.432
Hispanic	1418/9629 (12.0%)	1 (Ref)	0.87 (0.69, 1.09)	0.72 (0.57, 0.90)	0.72 (0.58, 0.90)	0.002		
Non-hispanic	3152/16031 (14.8%)	1 (Ref)	0.78 (0.67, 0.92)	0.66 (0.57, 0.78)	0.64 (0.54, 0.76)	<0.001		
Educational level:							0.583	0.648
Less than college	2585/12236 (17.7%)	1 (Ref)	0.81 (0.69, 0.95)	0.73 (0.61, 0.87)	0.70 (0.59, 0.83)	<0.001		
Some college or above	1985/13424 (12.0%)	1 (Ref)	0.78 (0.63, 0.97)	0.64 (0.53, 0.77)	0.62 (0.49, 0.77)	<0.001		
FPIR:							0.337	0.432
High	856/6370 (10.9%)	1 (Ref)	0.84 (0.64, 1.11)	0.69 (0.52, 0.91)	0.63 (0.46, 0.86)	0.002		
Low	1126/5833 (16.8%)	1 (Ref)	0.87 (0.67, 1.11)	0.76 (0.59, 0.98)	0.83 (0.64, 1.07)	0.083		
Middle	2588/13457 (15.8%)	1 (Ref)	0.75 (0.63, 0.89)	0.64 (0.55, 0.76)	0.62 (0.51, 0.75)	<0.001		
Marital status:							0.167	0.432
Live alone	2103/10306 (16.5%)	1 (Ref)	0.76 (0.62, 0.92)	0.60 (0.49, 0.73)	0.71 (0.58, 0.86)	<0.001		
Live together	2467/15354 (12.9%)	1 (Ref)	0.83 (0.70, 0.99)	0.72 (0.62, 0.85)	0.63 (0.52, 0.76)	<0.001		
Smoking status:							0.048	0.432
Current	799/5251 (12.2%)	1 (Ref)	0.72 (0.53, 0.98)	0.75 (0.55, 1.03)	0.78 (0.60, 1.03)	0.160		
Former	1486/6118 (19.0%)	1 (Ref)	0.65 (0.50, 0.85)	0.57 (0.46, 0.70)	0.50 (0.37, 0.68)	<0.001		
Never	2285/14291 (12.8%)	1 (Ref)	0.92 (0.77, 1.10)	0.72 (0.61, 0.86)	0.71 (0.59, 0.85)	0.002		
Drinking status:							0.338	0.432
Current	2940/18250 (12.6%)	1 (Ref)	0.78 (0.66, 0.93)	0.70 (0.59, 0.83)	0.65 (0.55, 0.77)	<0.001		
Former	771/3495 (19.5%)	1 (Ref)	0.84 (0.59, 1.19)	0.60 (0.42, 0.84)	0.51 (0.34, 0.74)	<0.001		
Never	859/3915 (19.4%)	1 (Ref)	0.86 (0.68, 1.07)	0.61 (0.48, 0.79)	0.88 (0.65, 1.21)	0.158		
Energy from Diet:							0.337	0.432
≤medium	2777/12835 (17.9%)	1 (Ref)	0.78 (0.67, 0.92)	0.71 (0.60, 0.83)	0.61 (0.51, 0.72)	<0.001		
>medium	1793/12825 (11.1%)	1 (Ref)	0.83 (0.67, 1.02)	0.65 (0.52, 0.81)	0.70 (0.57, 0.87)	<0.001		

All weighted ORs were estimated using multivariable logistic regression models with adjustment for survey cycles, sex, age, race, educational level, FPIR, marital status, smoking status, drinking status, BMI, hypertension, diabetes, dietary supplements, and dietary intake (protein, total fat, carbohydrate, and energy), except for the stratifying variable in each respective subgroup.

The values in the “Event/n” column represent the unweighted number of CKD cases and total participants. The percentages in parentheses indicate the weighted prevalence, accounting for the survey design.

All estimates were weighted to account for the complex, multistage sampling design of NHANES.

**Table 5:** Subgroup and interaction analyses of the association between LTPA and CKD risk in all qualified NHANES 2007-2016 participants.

		Four groups of LTPA					
Subgroup variable	Event/n (%)	Group 1	Group 2	Group 3	P _trend_	Raw P _interaction_	FDR-adjusted P _interaction_
survey year						0.894	0.935
2007–2010	1922/10687 (13.5%)	1 (Ref)	0.94(0.76, 1.16)	0.74(0.61, 0.88)	0.003		
2011-2016	2648/14973 (14.7%)	1 (Ref)	0.87(0.72, 1.05)	0.71(0.59, 0.85)	<0.001		
Sex:						0.242	0.539
Male	2148/12393 (12.5%)	1 (Ref)	0.80(0.67, 0.97)	0.69(0.59, 0.81)	<0.001		
Female	2422/13267 (15.8%)	1 (Ref)	0.96(0.82, 1.13)	0.74(0.59, 0.94)	0.012		
Age:						0.068	0.342
≤65	2166/20085 (8.9%)	1 (Ref)	0.94(0.78, 1.13)	0.60(0.51, 0.71)	<0.001		
>65	2404/5575 (43.1%)	1 (Ref)	0.68(0.56, 0.83)	0.57(0.45, 0.72)	<0.001		
Race:						0.538	0.770
Hispanic	1418/9629 (12.0%)	1 (Ref)	0.93(0.76, 1.15)	0.73(0.57, 0.93)	0.011		
Non-hispanic	3152/16031 (14.8%)	1 (Ref)	0.89(0.76, 1.03)	0.72(0.62, 0.84)	<0.001		
Educational level:						0.539	0.770
Less than college	2585/12236 (17.7%)	1 (Ref)	0.85(0.73, 1.00)	0.79(0.65, 0.97)	0.028		
Some college or above	1985/13424 (12.0%)	1 (Ref)	0.92(0.75, 1.12)	0.69(0.58, 0.83)	<0.001		
FPIR:						0.269	0.539
High	856/6370 (10.9%)	1 (Ref)	0.88(0.67, 1.15)	0.68(0.54, 0.87)	0.002		
Low	1126/5833 (16.8%)	1 (Ref)	1.02(0.79, 1.33)	0.92(0.71, 1.18)	0.494		
Middle	2588/13457 (15.8%)	1 (Ref)	0.87(0.74, 1.03)	0.70(0.57, 0.86)	<0.001		
Marital status:						0.645	0.806
Live alone	2103/10306 (16.5%)	1 (Ref)	0.84(0.69, 1.03)	0.72(0.59, 0.87)	0.001		
Live together	2467/15354 (12.9%)	1 (Ref)	0.93(0.78, 1.10)	0.72(0.61, 0.84)	<0.001		
Smoking status:						0.048	0.342
Current	799/5251 (12.2%)	1 (Ref)	0.94(0.67, 1.32)	0.75(0.54, 1.05)	0.094		
Former	1486/6118 (19.0%)	1 (Ref)	0.68(0.54, 0.85)	0.54(0.43, 0.68)	<0.001		
Never	2285/14291 (12.8%)	1 (Ref)	1.03(0.87, 1.22)	0.84(0.70, 1.01)	0.065		
Drinking status:						0.935	0.935
Current	2940/18250 (12.6%)	1 (Ref)	0.87(0.74, 1.02)	0.71(0.61, 0.84)	<0.001		
Former	771/3495 (19.5%)	1 (Ref)	0.94(0.66, 1.32)	0.68(0.48, 0.96)	0.030		
Never	859/3915 (19.4%)	1 (Ref)	0.99(0.75, 1.30)	0.76(0.54, 1.06)	0.101		
Energy from Diet:						0.194	0.539
≤medium	2777/12835 (17.9%)	1 (Ref)	0.82(0.69, 0.99)	0.76(0.62, 0.93)	0.007		
>medium	1793/12825 (11.1%)	1 (Ref)	0.99(0.84, 1.18)	0.69(0.57, 0.84)	0.005		

All weighted ORs were estimated using multivariable logistic regression models with adjustment for survey cycles, sex, age, race, educational level, FPIR, marital status, smoking status, drinking status, BMI, hypertension, diabetes, dietary supplements, and dietary intake (protein, total fat, carbohydrate, and energy), except for the stratifying variable in each respective subgroup.

The values in the “Event/n” column represent the unweighted number of CKD cases and total participants. The percentages in parentheses indicate the weighted prevalence, accounting for the survey design.

All estimates were weighted to account for the complex, multistage sampling design of NHANES.

## 4. Additional analysis

**Fig 2:**
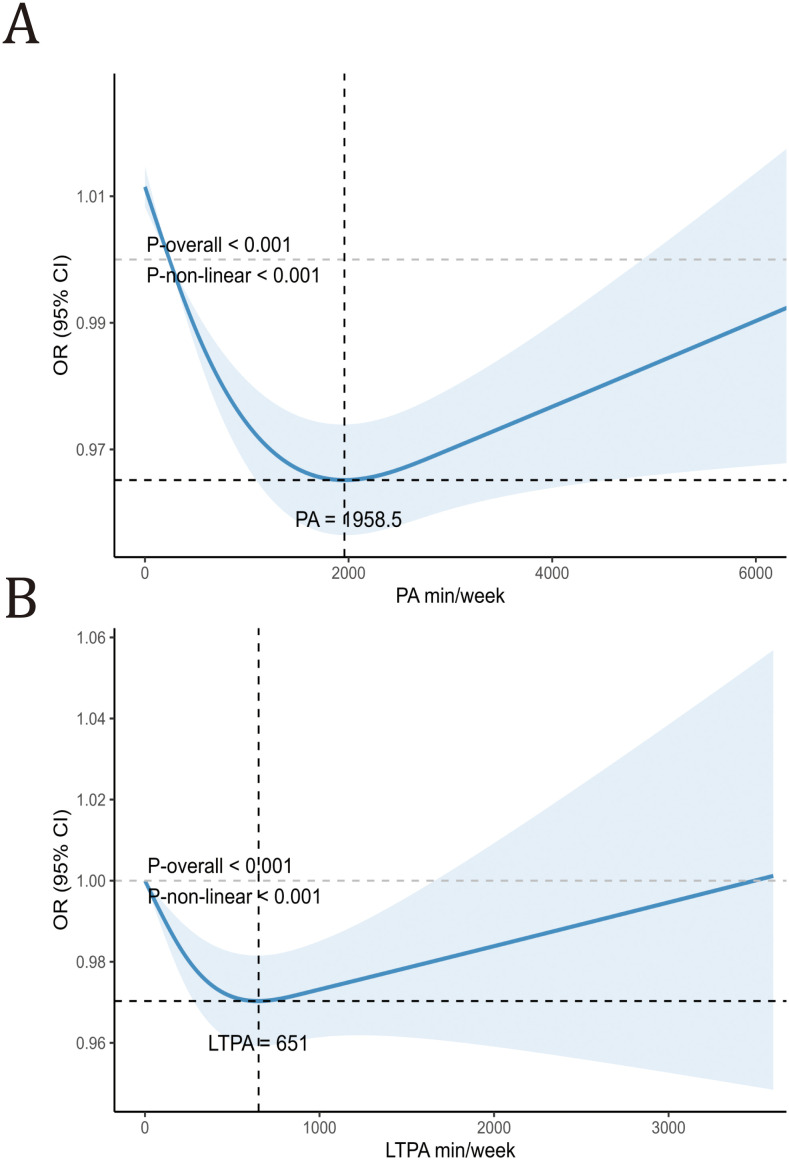
Dose-response relationship between total PA, LTPA, and CKD risk among NHANES 2007–2016 participants. Panel A shows the restricted cubic spline (RCS) curve for total PA and CKD risk, while Panel B illustrates the RCS curve for LTPA and CKD risk. All analyses were weighted and adjusted for survey cycles, sex, age, race, education level, FPIR, marital status, smoking status, alcohol consumption, dietary supplement, and dietary intake of protein, total fat, carbohydrates, and total energy.

To further explore the dose-response relationship between total PA, LTPA, and CKD, restricted cubic spline (RCS) models were employed. As shown in Fig 2, both total PA and LTPA demonstrated significant non-linear inverse associations with CKD incidence (P < 0.05 for non-linearity). A threshold effect was observed, with the protective effect of total PA reaching a plateau beyond approximately 1958.5 minutes/week, and that of LTPA beyond 651 minutes/week.

In addition, mediation analysis (Table 6) revealed that inflammatory markers, including WBC count and absolute neutrophil count mediated the association between total PA and CKD risk. Specifically, the average causal mediation effect (ACME) for WBC count was −0.000737 (95% CI: −0.001033 to −0.000422; P < 0.001), with a proportion mediated of 7.14% (95% CI: 3.25% to 12.90%; P < 0.001). For neutrophils, the ACME was −0.001023 (95% CI: −0.001303 to −0.000482; P < 0.001), with a proportion mediated of 9.89% (95% CI: 4.42% to 16.60%; P < 0.001).

**Table 6:** Mediation effects of potential mediators in the association between total PA and CKD risk.

Mediator	TOTAL effect	p	ACME	p	ADE	P	Proportion mediated	p
	Estimate (95% CI)		Estimate (95% CI)		Estimate (95% CI)		Estimate (95% CI)	
WBC	−0.010316(−0.016317, −0.005919)	<0.001	−0.000737(−0.001033, −0.000422)	<0.001	−0.009579(−0.015581, −0.005229)	<0.001	0.071400(0.032483, 0.129006)	<0.001
Neutrophils	−0.010335(−0.016073, −0.005582)	<0.001	−0.001023(−0.001303, −0.000482)	<0.001	−0.009312(−0.015103, −0.004800)	<0.001	0.098940(0.044158, 0.165999)	<0.001
Lymphocytes	−0.010671(−0.017319, −0.006125)	<0.001	0.000022(−0.000023, 0.000040)	0.842	−0.010693(−0.017326, −0.006129)	<0.001	−0.002064(−0.003964, 0.002045)	0.842
ALP	−0.010265(−0.017053, −0.005509)	<0.001	0.000002(−0.000277, 0.000057)	0.148	−0.010267(−0.016889, −0.005407)	<0.001	−0.000160(−0.006087, 0.030713)	0.148
BMI	−0.009823(−0.016446, −0.005276)	<0.001	−0.000930(−0.001047, −0.000471)	<0.001	−0.008893(−0.015712, −0.004549)	<0.001	0.094690(0.038471, 0.151659)	<0.001
Diabetes	−0.009273(−0.015484, −0.004732)	<0.001	−0.001124(−0.001652, −0.000886)	<0.001	−0.008149(−0.014145, −0.003429)	<0.001	0.121172(0.077782, 0.273245)	<0.001
Hypertension	−0.010124(−0.016475, −0.005261)	<0.001	−0.000784(−0.001093, −0.000262)	0.002	−0.009339(−0.015866, −0.004718)	<0.001	0.077467(0.021696, 0.132463)	0.002

Mediating analyses adjusted for survey cycles, sex, age, race, educational level, FPIR, marital status, smoking status, drinking status, dietary supplement, and dietary intake of protein, total fat, carbohydrate, and energy.

All estimates were weighted to account for the complex, multistage sampling design of NHANES.

Moreover, body mass index (BMI), diabetes, and hypertension were also found to mediate the relationship between total PA and CKD risk. The ACME for BMI was −0.000930 (95% CI: −0.001047 to −0.000471; P < 0.001), with a proportion mediated of 9.47% (95% CI: 3.85% to 15.17%; P < 0.001). For diabetes, the ACME was −0.001124 (95% CI: −0.001652 to −0.000886; P < 0.001), with a proportion mediated of 12.12% (95% CI: 7.78% to 27.32%; P < 0.001). The ACME for hypertension was −0.000784 (95% CI: −0.001093 to −0.000262; P = 0.002), with a proportion mediated of 7.75% (95% CI: 2.17% to 13.25%; P < 0.001).

## 5. Discussion

In the past, studies utilizing NHANES data have explored the relationship between PA and mortality rates among CKD patients within the overall population of the United States. Qu X et al. found that consistent PA can significantly reduce all-cause and specific-cause mortality rates among American CKD patients and can prevent disease progression in various ways [54]. Zhang NH et al. demonstrated that leisure-time PA can reduce the mortality rate of CKD and clinicians should encourage inactive CKD patients to perform PA [25]. Peng W et al. found that any level of PA was associated with reduced all-cause mortality in male CKD participants, whereas significant association was observed only in the extremely highly active group among female patients [55]. These studies have highlighted the contribution of PA in reducing mortality among CKD patients. However, no research has yet utilized NHANES data to investigate the impact of PA on the risk of CKD occurrence within the overall population of the United States. Therefore, we conducted this cross-sectional study using NHANES data to seek reliable evidence for the general population in the United States.

In this nationally representative cross-sectional survey of NHANES, we found that consistent total PA was associated with a reduced risk of CKD in the general population of the United States, regardless of survey cycles, age, sex, race, educational level, FPIR, marital status, smoking status, drinking status and dietary energy intake. Importantly, we also observed that leisure-time physical activity (LTPA) was independently associated with a lower risk of CKD, highlighting its potential role as a protective factor beyond total PA. Among the various PA domains analyzed, only LTPA showed a significant inverse relationship with CKD risk, suggesting that voluntary, self-directed, and sustainable forms of physical activity may have superior renal protective effects compared to occupational or transportation-related PA. Interestingly, we also observed that beyond a certain threshold, continued adherence to total PA and LTPA did not seem to confer additional benefits in terms of CKD risk.

Moreover, restricted cubic spline analyses revealed a non-linear dose-response relationship between total PA and CKD risk, with a plateau effect observed at approximately 1958.5 minutes/week for total PA and 651 minutes/week for LTPA. This suggests that while moderate increases in PA reduce CKD risk, excessively high levels may not provide further benefit.

To explore potential mechanisms, we conducted mediation analysis and identified five key mediators—white blood cell (WBC) count, neutrophil count, BMI, diabetes, and hypertension—that partially explained the relationship between total PA and CKD risk. These results suggest that PA reduces CKD risk not only directly but also indirectly by reducing systemic inflammation and improving metabolic health. Specifically, regular PA may alleviate low-grade inflammation, prevent obesity-related metabolic dysfunction, lower blood pressure, and reduce diabetes risk, all of which are established contributors to CKD onset.

As is well known, traditional risk factors for CKD, such as hypertension, diabetes, obesity, advanced age, and infections [19,56], are closely associated with long-term chronic low-grade inflammation. Chronic low-grade inflammation, characterized by a non-specific, persistent, and mild inflammatory state, is associated with an increased infiltration of inflammatory cells and cytokines, playing a crucial role as a risk factor for developing hypertension and diabetes [57]. Research has shown that the onset of chronic low-grade inflammation may be attributed to a lack of PA, resulting in the accumulation of visceral fat and subsequent increased infiltration of inflammatory immune cells, accompanied by an augmented release of adipokines [58]. In healthy adult subjects, appropriate PA has been shown to reduce the levels of systemic inflammatory markers, like C-reactive protein, interleukin (IL)-6, and tumor necrosis factor-α (TNF-α), while promoting the production of anti-inflammatory factors including IL-10, IL-12, IL-4, and transforming growth factor β-1 [59]. Additionally, regular PA enhances the function and abundance of regulatory T cells in peripheral blood [60], while decreasing the expression of Toll-like receptors in monocytes [61], resulting in suppressed pro-inflammatory cytokine release and increased anti-inflammatory cytokine secretion, thus mitigating systemic inflammation. However, lack of PA can trigger TLR signaling, leading to an increase in pro-inflammatory cytokine secretion and mediating systemic inflammatory responses [62]. Furthermore, research indicates that PA can participate in the regulation of TNF-α, IL-6, and interleukin-1 receptor antagonist (IL-1ra), thereby improving insulin resistance, enhancing insulin sensitivity, stimulating pancreatic B cell proliferation, and preventing pancreatic B cell damage, ultimately contributing to diabetes prevention [63]. Of note, engaging in PA has pronounced effects in delaying aging, reducing biological age, and preventing infections [64,65]. Skeletal muscle tissue is considered an endocrine organ capable of releasing a range of biologically active molecules, collectively referred to as myokines [66], offering a novel perspective for interpreting our research findings. For example, in a resting state, IL-6 mRNA levels in human muscle tissue are minimal. However, with prolonged PA and an increased number of contracting muscle fibers, skeletal muscle releases more IL-6, resulting in a nearly 100-fold increase in peripheral blood IL-6 concentration. Consequently, skeletal muscle is considered the primary tissue contributing to the elevation of circulating IL-6 levels [67]. IL-6 can mediate the anti-inflammatory effects of exercise by inhibiting the expression of the pro-inflammatory factor TNF-α in monocytes and promoting the upregulation of the anti-inflammatory factors IL-1ra and IL-10 in peripheral blood [68]. IL-15, a newly identified myokine, is prominently expressed in skeletal muscle during PA, effectively inhibiting fat accumulation, promoting fat breakdown, and reducing tissue inflammation [69,70]. Additionally, the other myokines synthesized by muscle tissue, such as brain-derived neurotrophic factor and insulin-like growth factor-1, also demonstrate the ability to suppress inflammatory responses [71–74].

Recent research has underscored that PA supports distinct therapeutic benefits, enhancing quality of life, cardiovascular health, and longevity; however, excessive PA may potentially mitigate these advantages [75]. Similarly, in this study, it was observed that beyond a certain threshold, continued PA did not yield significant benefits in lowering the risk of CKD. Several potential mechanisms might explain this phenomenon. It is well known that excessive PA can lead to rhabdomyolysis, lactate accumulation, and excessive purine production, increasing the burden on the kidneys and triggering renal inflammation, thereby counteracting the benefits of exercise [76,77]. Moreover, post-marathon athletes commonly experience an open window period, marked by a notable reduction in immune cells, such as lymphocytes and natural killer cells, suppressing immune function and response, consequently lowering immunity and significantly elevating the risk of infections [78,79]. Therefore, we hypothesize that prolonged PA participants may experience an open window period, disrupting immune balance, potentially triggering urinary tract infections, and affecting kidney structure and function. Additionally, a study identified a significant reduction in intrinsic mitochondrial function following a week of high PA load, potentially impacting renal tubular epithelial cells due to their high mitochondrial content and heavy energy demands [80,81].

Our study presents several strengths. This is the first investigation utilizing NHANES to explore the influence of PA on CKD risk. Furthermore, our research was based on the NHANES, a nationally representative cross-sectional survey of civilian, noninstitutionalized persons living in the USA. Therefore, our findings can be generalized to the U.S. population, regardless of age, sex, race, education level, FPIR, marital status, smoking status, drinking status and BMI. In addition, we found that both total PA and LTPA were independently associated with reduced CKD risk, and that the protective effect of PA plateaued at higher activity levels. This provides practical guidance for setting evidence-based physical activity goals for CKD prevention.

From a public health perspective, these findings underscore the importance of promoting regular physical activity, especially leisure-time physical activity, as a modifiable lifestyle factor in CKD prevention strategies. Given the increasing prevalence of CKD globally and its associated healthcare burden, integrating physical activity promotion into public health policies, primary care guidance, and community health programs could offer a cost-effective approach to reduce CKD incidence and progression. Our results also support the need for targeted interventions among high-risk groups to encourage sustainable physical activity patterns as part of chronic disease prevention and management frameworks.

However, our study has several limitations. Firstly, it is a cross-sectional study rather than a prospective one, thus unable to observe the effects of changes in individual PA habits on CKD risk. Secondly, the assessment of PA relied on self-report questionnaires rather than objective measurements and the collection of PA data was based on a typical week at a single point in time, failing to capture stable levels or trajectories of PA. Third, although our mediation analysis identified important pathways, residual confounding and measurement error cannot be fully ruled out. Fourth, despite finding that surpassing a specific total PA threshold doesn’t further reduce the risk of CKD, the threshold is quite high, and few individuals reach it. Hence, its guiding applicability is limited, but it remains clinically significant. Subsequent extensive research is essential to precisely determine this threshold.

## 6. Conclusions

In summary, this study identified significant non-linear inverse associations between total PA, LTPA, and the risk of CKD, with a threshold effect beyond which further increases in PA did not yield additional benefits. Notably, only LTPA showed an independent protective association with CKD, highlighting the importance of voluntary and sustainable forms of physical activity. Mediation analyses further suggested that these associations were partially explained by improvements in metabolic health and reductions in systemic inflammation. However, given the cross-sectional nature of this study, longitudinal studies are warranted to establish temporal relationships and further validate the causal mechanisms underlying the observed associations.

## Supporting information

S1 Fig Directed acyclic graph (DAG) illustrating hypothesized relationships between physical activity and chronic kidney disease. This Directed Acyclic Graph (DAG) visually illustrates the hypothesized causal relationships between physical activity (PA) and chronic kidney disease (CKD), as well as the identified confounders and mediators based on prior literature. The graph is used to guide the selection of appropriate covariates for multivariable logistic regression and mediation analysis in this study.
(TIF)

S2 Fig Overview of the statistical analysis workflow. This flowchart summarizes the overall data analysis process, including data preprocessing, multiple imputation, logistic regression, quartile-based analysis, restricted cubic spline modeling, subgroup and interaction analyses, and mediation analysis. All analyses accounted for NHANES complex survey design.
(TIF)

S1 Table Association between total PA, TPA, OPA and LTPA and odds of CKD in all qualified NHANES 2007–2016 participants. ^a^ Adjusted for survey cycles, sex, age, race, educational level, FPIR and marital status. ^b^ Adjusted for model 1 plus smoking status, drinking status, dietary supplements, and protein, total fat, carbohydrate and energy from diet. All data was weighted analysis based on the complex survey design of NHANES.
(DOCX)
