# Supplementary figures and images for: Non-linear associations of total and leisure-time physical activity with chronic kidney disease: Findings from NHANES
Source: PLoS One. 2025 Oct 8;20(10):e0334224. doi: 10.1371/journal.pone.0334224 (PMC12507227; doi:10.1371/journal.pone.0334224)

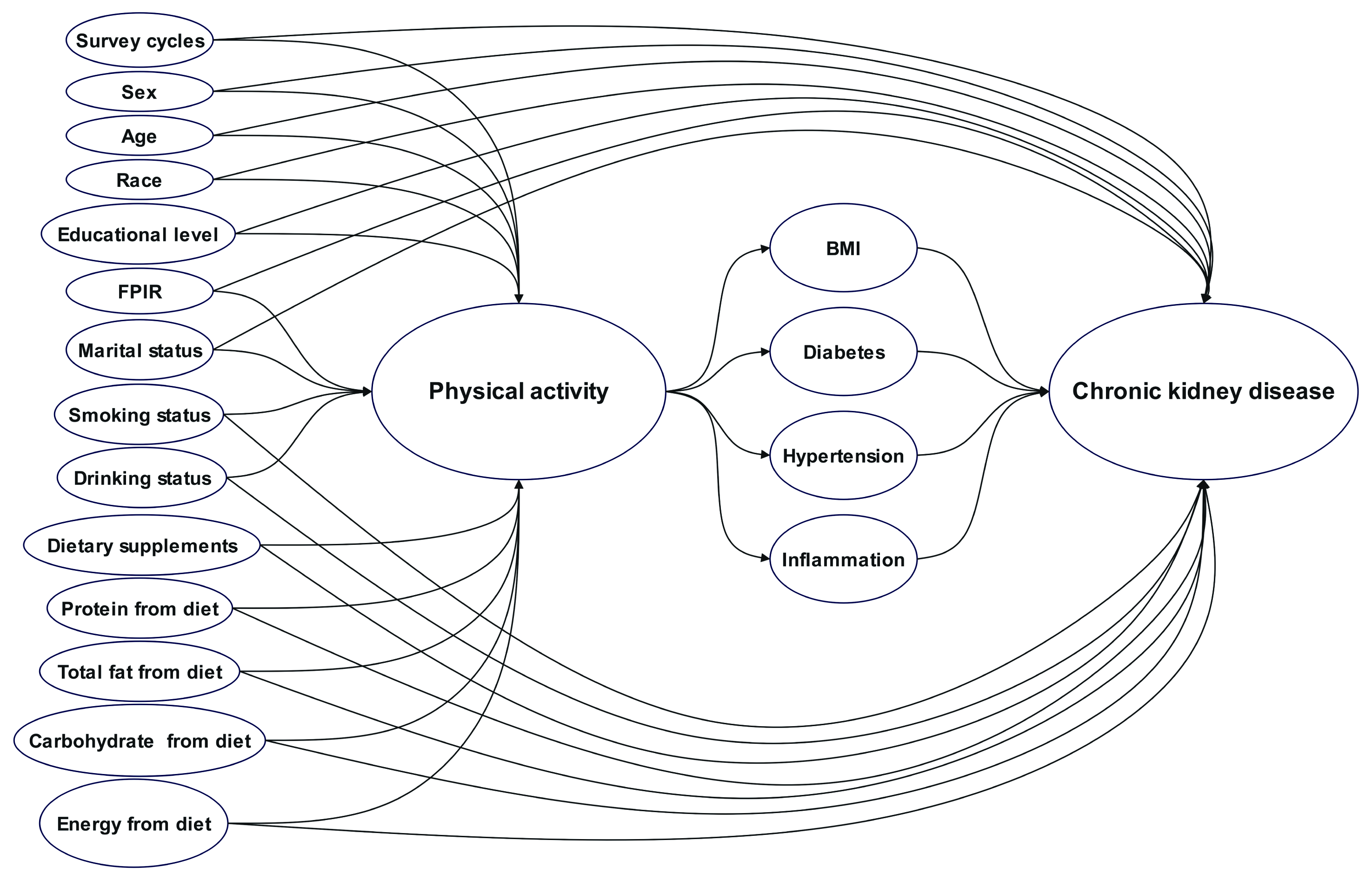

Supplement: S1 Fig — This Directed Acyclic Graph (DAG) visually illustrates the hypothesized causal relationships between physical activity (PA) and chronic kidney disease (CKD), as well as the identified confounders and mediators based on prior literature. The graph is used to guide the selection of appropriate covariates for multivariable logistic regression and mediation analysis in this study. (TIF) [file pone.0334224.s001.tif]

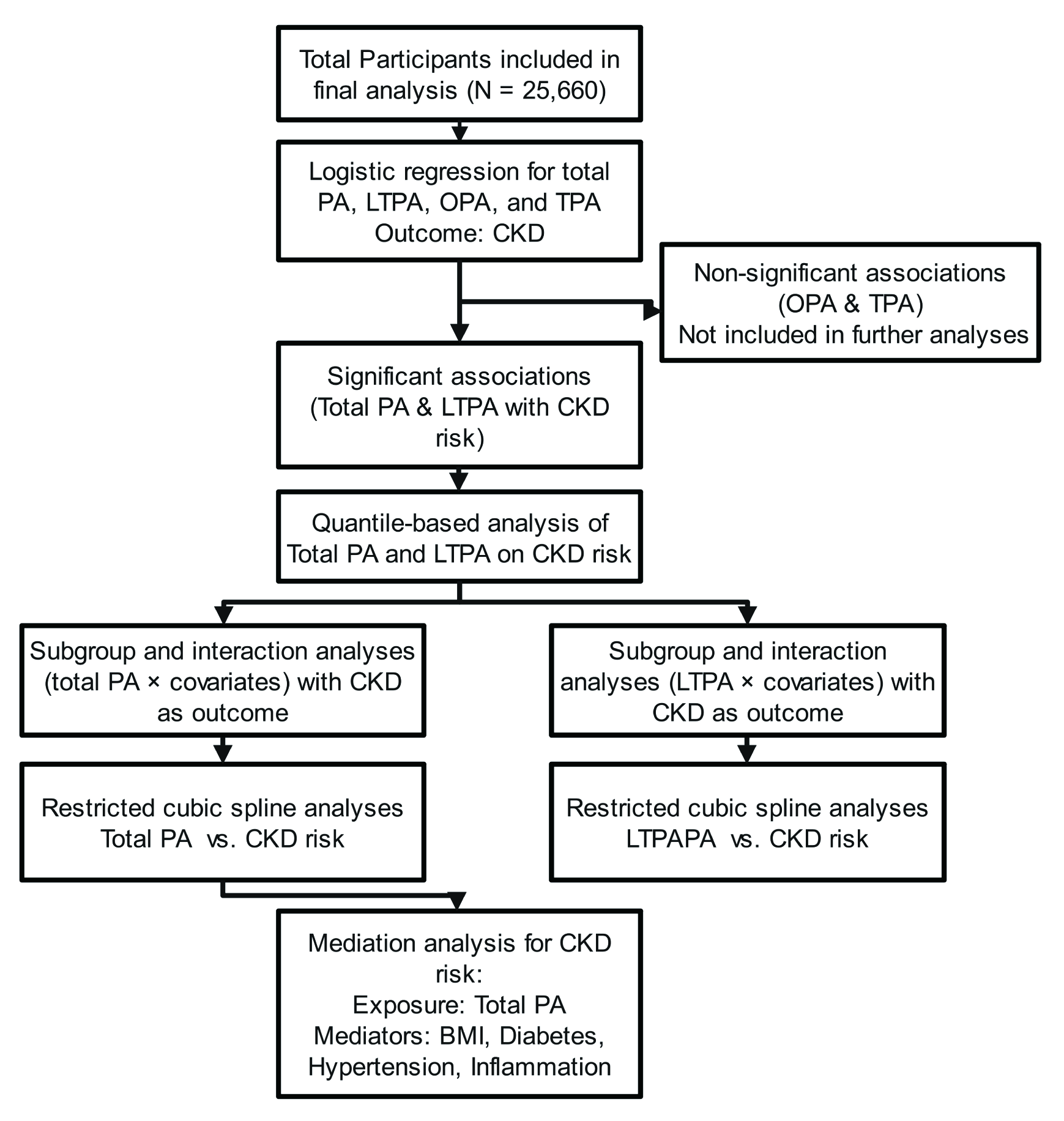

Supplement: S2 Fig — This flowchart summarizes the overall data analysis process, including data preprocessing, multiple imputation, logistic regression, quartile-based analysis, restricted cubic spline modeling, subgroup and interaction analyses, and mediation analysis. All analyses accounted for NHANES complex survey design. (TIF) [file pone.0334224.s002.tif]
