# Supplementary material for: Non-linear associations of total and leisure-time physical activity with chronic kidney disease: Findings from NHANES
Source: PLoS One. 2025 Oct 8;20(10):e0334224. doi: 10.1371/journal.pone.0334224 (PMC12507227; doi:10.1371/journal.pone.0334224)
Supplement: S1 Table — a Adjusted for survey cycles, sex, age, race, educational level, FPIR and marital status. b Adjusted for model 1 plus smoking status, drinking status, dietary supplements, and protein, total fat, carbohydrate and energy from diet. All data was weighted analysis based on the complex survey design of NHANES. (DOCX) [file pone.0334224.s003.docx]

**S1 table. Association between total PA, TPA, OPA and LTPA and odds of CKD in all qualified NHANES 2007-2016 participants.**

|  |  | **Unadjusted** | | **Model 1^a^** | | **Model 2^b^** | | |
| --- | --- | --- | --- | --- | --- | --- | --- | --- |
| **Per SD increment (min/week)** | **weighted means**  **(weighted SD)**  **(min/week)** | **OR (95% CI)** | **p** | **OR (95% CI)** | **p** | **OR (95% CI)** | **Raw P** | **FDR-adjusted P** |
| **Total PA** | 818.52(1,220.00) | 0.69(0.64, 0.74) | <0.001 | 0.89(0.83, 0.96) | 0.001 | 0.90(0.84, 0.96) | 0.003 | 0.006 |
| **TPA** | 74.99(258.78) | 0.87(0.82, 0.93) | <0.001 | 0.94(0.89, 1.00) | 0.061 | 0.94(0.89, 1.00) | 0.059 | 0.059 |
| **OPA** | 533.98(1,098.57) | 0.78(0.74, 0.84) | <0.001 | 0.93(0.87, 1.0) | 0.035 | 0.94(0.87, 1.00) | 0.053 | 0.059 |
| **LTPA** | 209.55(364.18) | 0.67(0.62, 0.73) | <0.001 | 0.87 (0.81, 0.94) | 0.001 | 0.89(0.82, 0.96) | 0.002 | 0.006 |

^a^ Adjusted for survey cycles, sex, age, race, educational level, FPIR and marital status.

^b^ Adjusted for model 1 plus smoking status, drinking status, dietary supplements, and protein, total fat, carbohydrate and energy from diet.

All data was weighted analysis based on the complex survey design of NHANES.

SD, standard deviation.
